# Child Opportunity Index Levels and Disparities in Access to Pediatric-ready Emergency Departments

**DOI:** 10.5811/westjem.53096

**Published:** 2026-05-19

**Authors:** Mary E. Bernardin, Paul Schuler, Emily Morales, Elizabeth Kendrick, Danielle Zoellner, Timothy Staed

**Affiliations:** * University of Missouri School of Medicine, Department of Emergency Medicine, Division of Pediatric Emergency Medicine; † University of Missouri School of Medicine, Department of Emergency Medicine, Division of Research

## Abstract

**Introduction:**

Increased pediatric readiness has been shown to decrease pediatric mortality, although disparities in access to pediatric-ready emergency departments (ED) have not been studied. The Child Opportunity Index (COI) is a comprehensive measure of the quality of neighborhood resources impacting child health and development. Our objective was to determine whether low-resourced areas with low COI levels are associated with farther travel distances to the nearest pediatric-ready ED.

**Methods:**

In this retrospective, cross-sectional study we evaluated the 2021 National Pediatric Readiness Project (NPRP) assessments of 91 EDs throughout the state of Missouri in relationship to COI 3.0 U.S. census tract data. The EDs were classified into quartiles based on weighted pediatric readiness scores (wPRS). Our primary outcome measure was travel distances to the nearest ED, which were obtained by measuring the shortest distance from the geographic center of each U.S. census tract to the closest ED. We used the Kruskal-Wallis H test to assess distances from the geographic center of each census tract to the nearest EDs. *P* values were adjusted for multiple comparisons using Dunn-Bonferroni post hoc tests.

**Results:**

Of the 113 EDs in Missouri that were invited to take the 2021 NPRP assessment, 91 (81%) participated and 22 (19%) were nonrespondent. Child Opportunity Index data were available for all 1,393 Missouri U.S. census tracts. When compared to low-resourced, low COI census tracts, well-resourced, very high COI census tracts were found to have significantly shorter travel distances to the nearest ED (6 vs 2.9 miles, [95% CI, 3.03–3.6; *P* < .001]). Families living in low COI census tracts travel 4.6 times farther (18 additional miles) to reach an ED in the highest wPRS quartile compared to families living in very high COI census tracts (23.3 vs 5.1 miles, [95% CI, 5.6–6.5; P < .001]). Families living in low COI census tracts travel 4.4 times farther (48 additional miles) to reach the nearest of the top three most pediatric-ready EDs [62.6 vs 14.4 miles, [95% CI, 14.6–19.1; P < .001].

**Conclusion:**

Families from resource-limited communities with low Child Opportunity Index levels must travel significantly farther to access pediatric-ready EDs compared to families from well-resourced communities. Dissemination of pediatric-readiness improvement efforts, especially to under-resourced areas, may help address disparities in healthcare access and promote health equity.

## INTRODUCTION

Increased pediatric readiness in emergency departments (ED) has been shown to significantly decrease the incidence of pediatric mortality and enhance quality of care with shorter hospital length of stay and fewer interfacility transfers.^1^–^5^ Iowa, Massachusetts, Nebraska, and New York, focusing on patients aged 0 to 18 years with critical illness, defined as requiring intensive care admission or experiencing death during the encounter. We used ED and inpatient administrative data from the Agency for Healthcare Research and Quality’s Healthcare Cost and Utilization Project linked to hospital-specific data from the 2013 National Pediatric Readiness Project. The relationship between hospital-specific pediatric readiness and encounter mortality in the entire cohort and in condition-specific subgroups was evaluated by using multivariable logistic regression and fractional polynomials. RESULTS: We studied 20 483 critically ill children presenting to 426 hospitals. The median weighted pediatric readiness score was 74.8 (interquartile range: 59.3–88.0; range: 29.6–100 Pediatric readiness efforts can include improvement in a range of areas impacting pediatric care, such as administration and care coordination, clinician competencies, equipment and supplies, quality improvement programs, policies, procedures and protocols.^6^ Although extensive efforts have been made to increase pediatric readiness through the National Pediatric Readiness Project (NPRP), many children lack access to pediatric-ready facilities.^7^–^9^ While most children have access to an ED within 30 minutes, only roughly one in three children can access an optimally pediatric-ready ED in the same timeframe.^7^ Proximity has been found to be the most important reason for ED selection in the case of a pediatric emergency, and children are most often transported to the nearest facility, even when alternative pediatric-ready EDs are also accessible.^8^

While access to pediatric-ready facilities varies geographically, so too does the overall health and wellness of the communities in which children are born and raised. The Child Opportunity Index (COI) comprehensively measures the quality of neighborhood resources and conditions impacting child wellbeing by assessing 44 community indicators across three domains: education; socioeconomic; and health and environmental.^10^ Children from less resourced, low COI communities have been found to experience a multiplicity of poor health outcomes when compared to children from high COI communities, including increased ED use,^11^ hospitalizations,^12^ need for critical care,^12^ and mortality.^12^,^13^ While the COI health and environment domain includes two indicators of healthcare resources (ie, percentage of individuals with health insurance coverage and density of nonprofit organizations providing health-related services), these do not include a measure of access to quality pediatric healthcare or pediatric-ready emergency care.^10^

Health disparities relating to pediatric readiness have been poorly studied. One study found that while children treated in the most pediatric-ready facilities had a significantly lower incidence of mortality, the greatest survival advantage relating to pediatric readiness was experienced by Black children.^14^ Given this, it was postulated that increasing pediatric readiness could be a means of promoting health equity.^14^,^15^ For example, the adoption of pediatric-specific trauma protocols and transfer policies may reduce disparities by standardizing pediatric care and reducing the effects of potential biases.^14^,^15^ However, this potential health promotion would only benefit those with access to pediatric-ready facilities.

**Population Health Research Capsule**

What do we already know about this issue?

*Higher pediatric ED readiness reduces child mortality, but disparities in access to pediatric-ready EDs are poorly understood*.

What was the research question?

*Are low Child Opportunity Index neighborhoods farther from pediatric-ready emergency departments?*

What was the major finding of the study?

*Families from low COI tracts travel farther to reach the most pediatric-ready EDs (23 vs 5 miles; p<0.001)*.

How does this improve population health?

*Identifying gaps in access to pediatric-ready EDs allows for targeted pediatric readiness expansion and promotion of health equity for all children*.

To evaluate access to pediatric-ready facilities from a health equity lens, we sought to assess travel distances to pediatric-ready EDs across the state of Missouri based on U.S. census tract COI levels. Access to pediatric-ready hospitals is known to have regional variation,^7^–^9^ although to our knowledge, it has not been studied relative to other social factors outside race/ethnicity.^14^ We hypothesized that socioeconomic and environmental factors may play a role in dictating a child’s access to pediatric-ready healthcare.

## METHODS

### Data Collection and Definitions

In this retrospective, cross-sectional study, we evaluated travel distances from U.S. census tracts across Missouri to surrounding EDs of varying pediatric readiness. Pediatric readiness was determined by the results of the 2021 National Pediatric Readiness Project (NPRP) assessment for eligible Missouri EDs. The quality of child opportunity by census tract was assessed using COI 3.0 data. This study follows the Strengthening the Reporting of Observational Studies in Epidemiology reporting guideline^16^ and was approved by the University of Missouri Institutional Review Board as exempt from the need for informed consent because it does not include human participants.

We obtained COI 3.0 data from DiversityDataKids.org for each U.S. census tract in the state of Missouri.^17^ While the exact number of children per census tract varies, we chose census tracts over ZIP codes due to their relatively consistent and homogenous population density.^18^ Compiled from 2013–2017 and published in 2024, COI 3.0 is the most recent version, which includes an expanded list of indicators relevant to child wellbeing. These indicators include 44 total factors within three domains (education, health and environment, and socioeconomic) and 14 subdomains.^10^ Indicators are measured for each U.S. census tract and converted to a z-score. Weighted averages are calculated from indicator z-scores for each of the three domains, and the domain scores are combined to produce the final COI.^10^ Our analyses of COI were performed by grouping census tracts by COI level. DataDiversityKids.org categorizes census tract data into five COI levels based on aggregated COI ranking against the national average: very low; low; moderate; high and very high. Very low is composed of the least resourced U.S. census tracts with the lowest COI scoring, and very high the most resourced, highest COI-scoring census tracts.^10^

We obtained census tract geographical shape files from the U.S. Census Bureau for each census tract in Missouri.^19^ We used the GeoPandas package to determine the centroid of each census tract shape, representing the geographical coordinate average for that census tract.^20^ Haversine distances, measuring the shortest distance from the centroid or geographic center of each census tract, were used to approximate the shortest travel distance to the nearest EDs.

We obtained a statewide report of Missouri hospitals eligible to participate in the 2021 NPRP Assessment from the Utah Data Coordinating Center.^21^ Hospitals were considered NPRP eligible and were invited to take the 2021 NPRP assessment if the facility had an ED that accepted patients 24 hours/day, 7 days/week, including general hospitals, children’s hospitals within a general hospital, stand-alone children’s hospitals, critical access hospitals, micro-hospitals, off-site hospitals or satellite EDs, and independently owned freestanding EDs. Participating hospitals received a weighted Pediatric Readiness Score (wPRS)—100 possible points based on self-assessment of staffing, resources, equipment, and transfer agreements.^22^,^23^ All NPRP eligible hospitals in the state of Missouri were classified into NPRP nonrespondents (quartile 0) or quartiles 1–4 based on wPRS (quartile 1 having the lowest level of pediatric readiness and quartile 4 the highest).

We obtained the geographical coordinates of each hospital from the Missouri Department of Health and Human Resources Time Critical Diagnosis Statewide System of Care map^24^ and then merged the coordinates with each NPRP hospital record. To assess access to emergency medical care, including the quality of pediatric readiness, we measured travel distances from each Missouri U.S. census tract centroid to the closest ED of each wPRS quartile. To assess access to the highest degree of pediatric readiness among Missouri EDs, we assessed travel distances from each census tract centroid to the top three pediatric-ready EDs in Missouri, each of which scored at least 95/100 on the 2021 NPRP Assessment.

### Data Analysis

We summarized continuous variables as means with standard deviations, and categorical variables as frequencies and percentages. Distances from U.S. census tract centroids were described in mean miles with standard deviations and median miles with interquartile ranges. We initially planned to use one-way analysis of variance (ANOVA) to test for differences in mean travel distances to EDs across COI groups. However, normality tests of residuals showed significant deviation from normal distribution across all models (Shapiro-Wilk *P* < .05). Therefore, we replaced ANOVA with the Kruskal-Wallis H test to assess differences in distribution of the nonparametric median distances from census tract centroid to the nearest EDs per COI level and wPRS quartiles. The generated *P* values were adjusted for multiple comparisons using Dunn-Bonferroni post hoc tests. We created box plots and maps using the Python packages Seaborn and Matplotlib. *P* values < .05 were considered statistically significant. We performed statistical analyses using Python software, v3.12.5.^25^

## RESULTS

Of the 113 hospitals in Missouri that were eligible to take the 2021 NPRP Assessment, 91 (81%) participated and 22 (19%) were nonrespondent. The average wPRS of participating Missouri hospitals was 66.55 of 100 possible points. The Missouri hospital response rate (80%) was 10% higher than the national average, while the average Missouri wPRS (66.55/100) was 2.95 points below the national average (69.5/100).^26^ Missouri’s highest scoring, quartile 4 hospitals scored on average 37.9 points higher than hospitals in quartile 1.

The COI data were available for all 1,393 Missouri U.S. census tracts, and all 1,393 tracts were included in analyses. The average COI for all available Missouri census tracts was 40.6 of 100 possible points. The most resourced, very high COI census tracts scored on average 78 points higher overall than those in the very low COI census tracts. Compared to the very low COI census tracts, census tracts in the very high COI group scored on average 70 points higher in the education domain, 64 points higher in the health and environment domain, and 75 points higher in the social and economic domain.^17^

**Figure 1:**
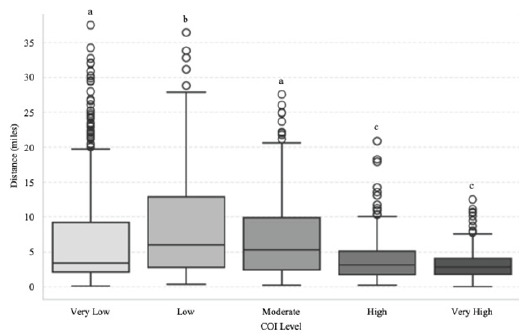
Box plot depicting travel distances to the nearest emergency department in relation to Child Opportunity Index level. Each box plot displays the median travel distance (the line inside the box) to the nearest emergency department by COI level, the interquartile (IQR) range of travel distances (the box), and the range of distances within 1.5 times the IQR (the whiskers), with dots representing outliers. compared to the less resourced, very low, low, and moderate COI census tracts. *COI*, Child Opportunity Index.

Census tract COI was found to be significantly associated with travel distances to the nearest ED. Post hoc multigroup comparisons of travel distances to the nearest EDs are depicted in Table 1 with corresponding box plots in Figure 1. The most resourced, very high COI census tracts were found to have significantly shorter travel distances to the nearest ED when compared to the less resourced, very low, low, and moderate COI census tracts (*P* < .001). Families living in low COI census tracts travel 2.1 times farther (3.2 additional miles) than families living in the very high COI census tracts (*P* < .001) to reach the nearest ED.

**Figure 2:**
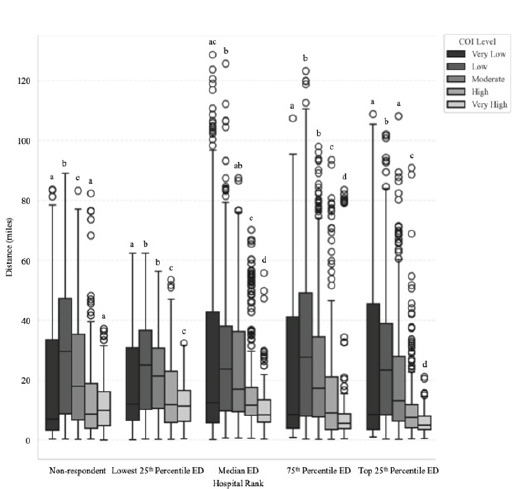
Travel distances in miles to the nearest emergency department by Child Opportunity Index level and weighted Pediatric Readiness Score quartiles. Each box plot displays the median travel distance (the line inside the box) to the nearest emergency department by Child Opportunity Index level and wPRS hospital ranking, the interquartile range (IQR) of travel distances (the box), and the range of distances within 1.5 times the IQR (the whiskers), with dots representing outliers. *ED*, emergency department.

U.S. census tract COI levels were found to have significant differences in travel distances to the nearest ED when categorized by wPRS quartiles. Post hoc multigroup comparisons of travel distances to the nearest ED by COI level among wPRS quartiles are depicted in Table 2 with corresponding box plots in Figure 2. Families living in well-resourced, very high COI census tracts had significantly shorter travel distances to both nonrespondent EDs (wPRS quartile 0) as well as to EDs at each level of pediatric readiness by wPRS quartile when compared to all of the less resourced, lower COI levels. Families living in low resourced, low COI census tracts travel 4.6 times farther (18 additional miles) to reach an ED in the highest pediatric readiness quartile compared to families living in the most resourced, very high COI census tracts (*P* < .001).

**Figure 3:**
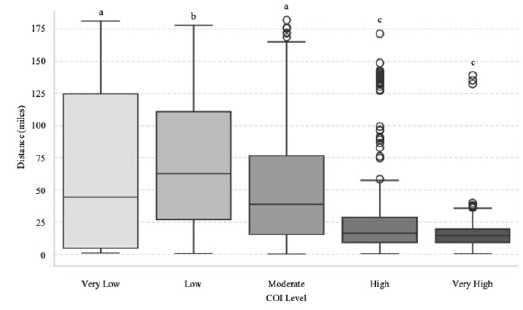
Travel distances in miles to the nearest of the top 3 weighted Pediatric Readiness Score emergency departments in the state of Missouri by Child Opportunity Index level. Each box plot displays the median travel distance (the line inside the box) to the nearest of the top 3 wPRS EDs by COI level, the interquartile range of travel distances (the box), and the range of distances within 1.5 times the interquartile range (the whiskers), with dots representing outliers. *COI*, Child Opportunity Index; *ED*, emergency department; *wPRS*, weighted Pediatric Readiness Score.

**Figure 4:**
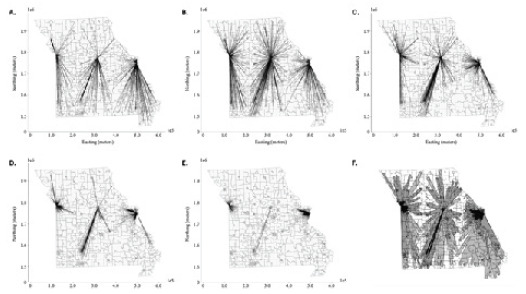
Travel distances in miles to the nearest of the top three weighted Pediatric Readiness Score emergency departments in Missouri by Child Opportunity Index (COI) level. A. Travel distances for very low COI census tracts. B. Travel distances for low COI census tracts. C. Travel distances for moderate COI census tracts. D. Travel distances for high COI census tracts. E. Travel distances for very high COI census tracts. F) Composite map showing low (light gray), moderate (dark gray), and very high (black) COI census tracts.

When assessing travel distances to the closest of the top three wPRS EDs, we found significant differences based on COI level. Post hoc multigroup comparisons of travel distances to the nearest of the top three pediatric ready EDs are depicted in Table 3 with corresponding box plots in Figure 3. Figure 4 visually depicts travel distances across Missouri to the nearest of the top three wPRS EDs by COI level. Families living in well resourced, very high COI U.S. census tracts had significantly shorter travel distances to the nearest of the top three wPRS EDs when compared to less resourced, very low, low, and moderate COI census tracts (*P* < .001). Families living in low COI census tracts travel 4.4 times farther (48 additional miles) to reach the nearest of the top three pediatric-ready EDs in Missouri compared to families living in very high COI census tracts (*P* < .001).

Figure 3 visually depicts shorter travel distances to the nearest of the top three wPRS EDs for families living in well resourced, very high COI census tracts when compared to less resourced, very low, low, and moderate COI census tracts.

## DISCUSSION

Momentous nationwide emphasis has been placed on advancing pediatric readiness through the NPRP, as increased pediatric readiness has been shown to significantly decrease the incidence of child mortality.^1^–^5^ Thus far, the majority of research involving pediatric readiness has understandably focused on associated health outcomes.^1^–^4^,^27^ Aside from evaluation of race/ethnicity, this is the first study to our knowledge that evaluates access to pediatric-ready healthcare in terms of social disparities affecting childhood opportunity. Using the COI, a comprehensive index measuring multiple factors impacting child health and wellbeing, we assessed travel distances to both the nearest ED and to a gradient of the closest pediatric-ready facilities. Our study revealed that when compared to children from highly resourced, high COI communities, children from less resourced, low COI communities have poorer access to any ED and significantly poorer access to pediatric-ready facilities. Our findings highlight the need for pediatric-readiness improvement efforts for all EDs to address health disparities and promote equitable healthcare access for all children.

Previous studies have demonstrated geographic varition in pediatric readiness.^7^–^9^ Ray et al found that access to pediatric ready facilities varied across U.S. census divisions, with Missouri in the West North Central group having the poorest access to EDs ≥ the 75^th^ percentile of pediatric readiness scores.^7^ While this established that access to pediatric ready facilities varies on a large, regional level, our findings suggest that access also varies at the U.S. census tract level and is associated with the existence of community resources. Other studies have found the largest deficit in access to pediatric ready EDs exists in rural regions.^8^,^9^ In our study, low COI census tracts were associated with poorer access to pediatric-ready EDs in both rural and urban settings, suggesting that lack of pediatric-ready healthcare may be more closely related to socioeconomic disadvantage rather than a function of rurality or urbanicity. Further studies are needed to investigate inequity in healthcare access as it relates to pediatric readiness across varying geographic and socioeconomic landscapes.

While travel distance or time are generally used to measure geographic “accessibility,” those integers do not account for all variables impacting a family’s ability to access pediatric-ready healthcare.^7^ A family may be unable to traverse even relatively short travel distances if they lack a reliable family vehicle or the financial means to purchase gas. Similarly, a parent’s choice for their child’s healthcare may be limited to whatever facility is most accessible by public transportation. Families with no means of travel may resort to requesting ambulance services, although their child may be transported to the closest ED despite more pediatric-ready facilities being accessible via emergency medical services (EMS).^7^,^8^ Studies show that social factors including unknown insurance status, low income, and communication barriers have been associated with higher likelihood of a child being transported by EMS to a general ED instead of a children’s ED.^28^ Healthcare accessibility is a complex, multifaceted issue, and increasing access to pediatric-ready EDs requires a multifaceted approach. Increasing the level of EMS responders’ education about local pediatric-ready facilities and the utility of transport to such facilities, including protocols for bypassing closer, less pediatric-ready general EDs, is one implementable approach.^7^

Our study revealed that children from under-resourced, low COI communities not only have significantly farther travel distances to pediatric-ready facilities compared to high COI communities, but they also have farther travel distances to the nearest ED, regardless of pediatric readiness. While some may infer that the necessary first step in addressing this issue is the distribution of new healthcare facilities into disadvantaged areas, research has shown that this is an unlikely solution. New trauma centers, for example, are most frequently established in highly populated, high median income locations.^29^ Thus, in the absence of new healthcare institutions, it becomes crucially important that the existing facilities, including those with historically low pediatric patient volumes, enhance their pediatric readiness.^9^ While low pediatric volume EDs tend to have lower wPRS,^5^ research has shown that participating in state-led, pediatric-readiness verification programs has been associated with significant increases in wPRS and subsequently decreased incidence of pediatric mortality.^30^,^31^ For this reason, it has been proposed that disseminating state-led pediatric readiness verification programs is of utmost importance in under-resourced areas with the greatest disparities in access to pediatric-ready EDs.^7^

Using public health policy is another effective means of promoting pediatric readiness. For instance, Illinois exemplifies government commitment to pediatric readiness by requiring that hospitals providing prehospital medical oversight participate in their state-wide pediatric facility recognition program. As a result, 60% of EDs in Illinois have been recognized by the Illinois Department of Public Health as pediatric ready, and those recognized as pediatric ready have exhibited a decrease in the incidence of pediatric injury mortality from 12.2 to 9/1,000 patients.^32^ The findings of our study and similar studies critically analyzing healthcare access can be used to inform public health policy and promote investment in pediatric readiness, particularly by expanding pediatric facility recognition efforts in areas experiencing the greatest inequity.^7^

Previous work has found increasing pediatric readiness to be associated with increased incidence of child survival, particularly among Black children.^14^ This finding led to the hypothesis that increased pediatric readiness, likely via increased standardization of care with pediatric treatment protocols, may be a means of addressing health inequity.^14^,^33^ However, it is also known that health-promoting activities can broaden existing health disparities because participation in these activities is often greatest among communities that already experience economic and health advantages.^34^ Because the highly resourced, high COI communities in our study were found to have significantly greater access to pediatric-ready EDs, our findings could be interpreted as evidence that NPRP efforts are already predominantly benefitting privileged groups and, hence, are promoting rather than alleviating health disparities.

An actionable solution to confront this inequity is targeted dissemination of pediatric-readiness improvement efforts to under-resourced areas that have not yet engaged in a pediatric-readiness improvement process. Public policy allocating resources that support state-led pediatric readiness programs throughout under-resourced areas is an essential investment in the health of historically disinvested communities.^7^ As research shows that actions improving the health of children promote the health of the future adult population,^35^ targeted investment in pediatric-ready healthcare among disinvested communities may serve as means of promoting health equity for future generations.

## LIMITATIONS

This study must be interpreted considering multiple potential limitations. Our assessment of access to pediatric-ready EDs was only carried out in the state of Missouri, and our results may not be applicable to other states and countries outside the United States. We did not assess travel distances to EDs outside the state of Missouri; therefore, lack of data incorporating travel across state lines could have impacted our findings. To measure travel distances to the nearest EDs, we calculated and used the centroid of each U.S. census tract, representing the geographical average coordinate. This estimate does not represent true travel distances for each household, nor does it take into account traffic, construction, or other environmental or social barriers to travel, which could impact the study’s findings.

We did not incorporate U.S. Census Bureau data regarding the exact number of children living in each census tract, although we chose census tracts over ZIP codes because of their homogenous population density.^18^ We calculated the wPRS based on a hospital’s self-assessment, which allowed for the potential of bias or inaccurate representation of EDs among wPRS quartiles. Additionally, 1 of the 44 indicators comprised in the COI 3.0 is a measure of the density of nonprofit organizations providing health-related services. Because of this, there is the potential that our findings could have been skewed by increased access to general health services as the COI level increases. However, density of nonprofit organizations providing health services in a community does not equate to pediatric-ready emergency care, and the quality of healthcare accessible to children is not currently represented in the COI.

Finally, our assertion that increasing pediatric readiness in under-resourced areas may improve health outcomes (eg, lower incidence of mortality) is complex and assumes that pediatric readiness is the primary driver of such outcomes. However, the relationship between low ED pediatric readiness and higher child mortality may be confounded by the general health of children accessing these EDs, if EDs with low pediatric readiness are most accessible to children of low COI level communities who may be independently at higher risk for poorer outcomes. Nonetheless, advanced pediatric readiness has been shown to decrease the incidence of child mortality,^1^ making efforts to improve pediatric readiness an actionable process that could improve outcomes and combat health disparities among high-risk, under-resourced communities.

## CONCLUSION

In this state-wide study in Missouri, children from under-resourced communities with low Childhood Opportunity Index levels were found to have significantly farther travel distances to the nearest ED as well as to surrounding pediatric-ready facilities. These findings highlight the crucial importance of increasing pediatric-readiness efforts for all EDs, especially those serving under-resourced areas. This and future research on disparities in healthcare access may inform public health policy and targeted investment in healthcare programs dedicated to improving pediatric readiness and promoting health equity.

## Figures and Tables

**Table 1:** Travel distances to nearest emergency department in study assessing the relationship between distances traveled and Child Opportunity Index level.

COI level	Mean (miles) (SD)	Median (miles)	95% Confidence interval	Bonferroni comparison	*P* value
Very low	7.34 (8.20)	3.41	6.44 – 8.23	-	-
Low	8.68 (7.27)	6.02	8.01 – 9.34	Low vs Very Low	< .001
Moderate	6.86 (5.49)	5.28	6.30 – 7.41	Moderate vs Very Low	.31
				Moderate vs Low	.04
High	3.94 (3.08)	3.12	3.58 – 4.30	High vs Very Low	.01
				High vs Low	< .001
				High vs Moderate	< .001
Very high	3.32 (2.16)	2.85	3.03 – 3.61	Very High vs Very Low	< .001
				Very High vs Low	< .001
				Very High vs Moderate	< .001
				Very High vs High	1.00

*SD*, standard deviation.

**Table 2:** Travel distances to nearest emergency department by Child Opportunity Index level among weighted Pediatric Readiness Score quartiles.

wPRS Quartile	COI Level	Mean (miles) (SD)	Median (miles)	95% Confidence Interval	Bonferroni comparison	*P* value
0	Very Low	19.62 (22.20)	7.00	17.19 – 22.05	-	-
	Low	30.73 (22.32)	29.60	28.69 – 32.77	Low vs Very Low	< .001
	Moderate	23.87 (20.35)	18.01	21.81 – 25.94	Moderate vs Very Low	< .001
					Moderate vs Low	.001
	High	13.37 (13.44)	9.06	11.79 – 14.94	High vs Very Low	1.000
					High vs Low	< .001
					High vs Moderate	< .001
	Very High	11.48 (8.22)	9.5	10.36 – 12.59	Very High vs Very Low	.93
					Very High vs Low	< .001
					Very High vs Moderate	< .001
					Very High vs High	1.0
1	Very Low	19.55 (16.04)	11.96	17.79 – 21.31	-	-
	Low	24.79 (15.42)	25.03	23.38 – 26.19	Low vs Very Low	< .001
	Moderate	21.81 (13.28)	21.28	20.46 – 23.15	Moderate vs Very Low	.01
					Moderate vs Low	.49
	High	14.69 (10.80)	11.76	13.43 – 15.95	High vs Very Low	.02
					High vs Low	< .001
					High vs Moderate	< .001
	Very High	12.48 (8.20)	11.30	11.36 – 13.59	Very High vs Very Low	< .001
					Very High vs Low	< .001
					Very High vs Moderate	< .001
					Very High vs High	1.0
2	Very Low	26.87 (29.55)	12.47	23.63 – 30.10	-	-
	Low	27.07 (21.57)	23.63	25.10 – 29.04	Low vs Very Low	< .001
	Moderate	23.86 (18.78)	16.98	21.95 – 25.77	Moderate vs Very Low	.07
					Moderate vs Low	1.0
	High	16.96 (14.98)	11.62	15.20 – 18.71	High vs Very Low	1.0
					High vs Low	< .001
					High vs Moderate	< .001
	Very High	10.35 (7.84)	8.50	9.28 – 11.41	Very High vs Very Low	< .001
					Very High vs Low	< .001
					Very High vs Moderate	< .001
					Very High vs High	< .001
3	Very Low	24.07 (25.59)	8.59	21.27 – 26.87	-	-
	Low	32.19 (27.22)	27.69	29.70 – 34.67	Low vs Very Low	< .001
	Moderate	25.33 (23.26)	17.27	22.97 – 27.69	Moderate vs Very Low	.02
					Moderate vs Low	.06
	High	16.17 (19.40)	9.06	13.90 – 18.44	High vs Very Low	.04
					High vs Low	< .001
					High vs Moderate	< .001
	Very High	9.11 (14.21)	5.54	7.18 – 11.04	Very High vs Very Low	< .001
					Very High vs Low	< .001
					Very High vs Moderate	< .001
					Very High vs High	< .001
4	Very Low	25.27 (27.37)	8.59	22.28 – 28.27	-	-
	Low	27.49 (22.08)	23.27	25.47 – 29.50	Low vs Very Low	< .001
	Moderate	19.85 (19.04)	13.15	17.92 – 21.79	Moderate vs Very Low	1.00
					Moderate vs Low	< .001
	High	10.67 (12.13	7.54	9.25 – 12.09	High vs Very Low	< .001
					High vs Low	< .001
					High vs Moderate	< .001
	Very High	6.04 (3.60)	5.07	5.55 – 6.53	Very High vs Very Low	< .001
					Very High vs Low	< .001
					Very High vs Moderate	< .001
					Very High vs High	.01

*ED*, emergency department; *COI*, Child Opportunity Index; *wPRS*, weighted Pediatric Readiness Score; *SD*, standard deviation.

**Table 3:** Travel distances to the nearest top three weighted Pediatric Readiness Score emergency departments in the state of Missouri by Child Opportunity Index level.

COI Level	Mean (miles) (SD)	Median (miles)	95% Confidence Interval	Bonferroni comparison	*P* value
Very Low	60.08 (59.71)	44.42	53.55 – 66.20	-	-
Low	71.26 (50.86)	62.64	66.62 – 75.91	Low vs Very Low	< .001
Moderate	53.44 (47.17)	38.77	48.65 – 58.23	Moderate vs Very Low	.12
				Moderate vs Low	< .001
High	30.57 (38.87)	16.49	26.02 – 35.12	High vs Very Low	< .001
				High vs Low	< .001
				High vs Moderate	< .001
Very High	16.86 (16.52)	14.40	14.61 – 19.10	Very High vs Very Low	< .001
				Very High vs Low	< .001
				Very High vs Moderate	< .001
				Very High vs High	.18

*COI*, Child Opportunity Index; *SD*, standard deviation.
